# The effect of self-sampled HPV testing on participation to cervical cancer screening in Italy: a randomised controlled trial (ISRCTN96071600)

**DOI:** 10.1038/sj.bjc.6606040

**Published:** 2010-12-21

**Authors:** P Giorgi Rossi, L M Marsili, L Camilloni, A Iossa, A Lattanzi, C Sani, C Di Pierro, G Grazzini, C Angeloni, P Capparucci, A Pellegrini, M L Schiboni, A Sperati, M Confortini, C Bellanova, A D'Addetta, E Mania, C B Visioli, E Sereno, F Carozzi, the Self-Sampling Study Working Group

**Affiliations:** 1 Agency for Public Health, Lazio Region, Via di S Costanza 53, Rome 00198, Italy; 2 Local Health Unit ASL RMC, Via Monza 2A, Rome 00182, Italy; 3 ISPO, Cancer Prevention and Research Institute, Viale A Volta 171, Florence 50131, Italy; 4 Coordinamento Programmi di Screening Regione Abruzzo, Ospedale di Atri, Italy; 5 San Giovanni Hospital, Via dell’Amba Aradam 9, Rome 00184, Italy

**Keywords:** Pap-test, HPV test, screening programme, cervical cancer prevention

## Abstract

**Background:**

In Italy, cervical cancer screening programmes actively invite women aged 25–64 years. Programmes are hindered by low participation.

**Methods:**

A sample of non-responder women aged 35–64 years, belonging to three different programmes (in Rome, Florence and Teramo), was randomly split into four arms: two control groups received standard recall letters to perform either Pap-test (first group) or human papillomavirus (HPV) test (second group) at the clinic. A third arm was sent letters offering a self-sampler for HPV testing, to be requested by phone, whereas a fourth group was directly sent the self-samplers home.

**Results:**

Compliance with standard recall was 13.9% (N619). Offering HPV test at the clinic had a nonsignificant effect on compliance (N616, relative risk (RR)=1.08; 95% CI=0.82–1.41). Self-sampler at request had the poorest performance, 8.7% (N622, RR=0.62; 95% CI=0.45–0.86), whereas direct mailing of the self-sampler registered the highest compliance: 19.6% (N616, RR=1.41; 95% CI=1.10–1.82). This effect on compliance was observed only in urban areas, Florence and Rome (N438, RR=1.69; 95% CI=1.24–2.30), but not in Abruzzo (N178, RR=0.95; 95% CI=0.61–1.50), a prevalently rural area.

**Conclusions:**

Mailing self-samplers to non-responders may increase compliance as compared with delivering standard recall letters. Nevertheless, effectiveness is context specific and the strategy costs should be carefully considered.

Testing for human papillomavirus (HPV) DNA in the cervix has been shown to be an effective mean for cervical cancer prevention in large population trials carried out in different countries with huge differences in social, cultural and economical background (Bulkmans *et al*, 2007; Naucler *et al*, 2007; Sankaranarayanan *et al*, 2009; Ronco *et al*, 2010). Human papillomavirus test is more sensitive than conventional Pap-test (Cuzick *et al*, 2006, 2008), giving a longer protection in case of negative results, and the overdiagnosis can be controlled at least in women aged 35 years and older (Bulkmans *et al*, 2007; Naucler *et al*, 2007; Sankaranarayanan *et al*, 2009; Ronco *et al*, 2010).

Even if an increase in test sensitivity is welcome, this is not the main problem in cervical cancer prevention. In fact, in most industrialised countries, the majority of invasive cancers occurs in never-screened or under-screened women (van der Graaf *et al*, 1988; Ciatto *et al*, 1993; Janerich *et al*, 1995; Sasieni *et al*, 1996; Sung *et al*, 2000; Leyden *et al*, 2005; Morrell *et al*, 2005; Bos *et al*, 2006; Ingemann-Hansen *et al*, 2008). As a consequence, the heaviest and most immediate impact on cervical cancer prevention can be obtained only by improving the test coverage (IARC, 2005; Arbyn *et al*, 2008).

In Italy, more than two-thirds of the female population aged 25–64 years is covered by screening programmes that actively invite the whole target population by mail every 3 years. Compliance with invitation is low, about 40% (Ronco *et al*, 2008). Nevertheless, the test coverage is estimated to be quite high, >70% (data from National Health Interview (ISTAT, 2006) and PASSI study (PASSI, 2009)), owing to opportunistic testing by private and public gynaecologists. Furthermore, wide variations in test coverage are observed among regions, ranging from 48 to 88% (ISTAT, 2006).

The use of HPV DNA test as primary screening test allows for the introduction of self-sampling (Wright *et al*, 2000; Nobbenhuis *et al*, 2002; Sanner *et al*, 2009; Gök *et al*, 2010), whereas conventional Pap-test requires sampling to be performed by a health professional. The opportunity of a home, self-collected sample, opens the chance to remove some of the barriers that may discourage women from participating to screening programmes or performing Pap-test. Self-sampling is less time consuming and invasive as compared with tests performed at a clinic. It allows for privacy, reduces discomfort and women know nobody would have to handle their body.

The objective was to measure the effect on test compliance of introducing a self sampler device using different strategies.

## Population and methods

### Setting

The study was performed within three organised screening programmes from three different Italian regions: Florence (Tuscany), Rome southern city (Lazio) and Teramo (Abruzzo). In these areas, programmes actively invite all the resident female population aged 25–64 years for a Pap-test every 3 years.

### Study population

Women aged 35–65 years who had been invited by the screening programme in the previous months and had failed to respond were eligible for mail recall. Each programme has a different strategy for recall: in Florence, recalls are sent to all non-responding women after invitations for a whole district are completed, which is to say after a period of at least 3 months and not more than 5 months. In Rome and Teramo, recalls are automatically mailed to non-responders 3 months after the invitation.

### Study design

**Figure 1:**
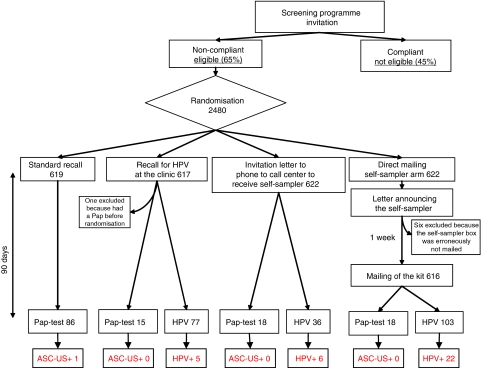
Flowchart of the study design. ASC-US+, women with cytology atypical squamous cells of unknown significance or more severe; HPV+, women with high-risk HPV DNA test positive.

A random sample of 2480 eligible women was randomly assigned to one of the following arms (Figure 1):

1. two control arms with a standard invitation letter to perform either a Pap-test (a) or an HPV test (b) at the clinic on a pre-fixed date;
2. two intervention arms:
  1. a group was offered the opportunity to receive the self-sampler device (PantaRhei Devices, Zeist, the Netherlands) (Brink *et al*, 2006) (by mail or picking it up at the clinic). If interested, women had to call a free toll number.
  2. another group was directly sent the self-sampler, announced by a letter a week before.

The lists of eligible women were provided by the centres. When known, women of the same household were identified with a flag and randomised as a unique item to the same arm (we decided not to account for this in the analysis owing to the extremely low number of these couples). Random sampling and arm assignment were performed centrally by the coordinating centre (Rome) using STATA 8.2 (StataCorp, College Station, TX, USA) and putting as seed for random number generator the first number drawn by the most recent National Lottery: four consecutive samples of the pre-determined size of independent statistical units were drawn and assigned to the corresponding arm. Couples of women resident in the same address were randomised as a single statistical unit.

The planned sample size was 200 women per arm per centre. This would allow for 80% power to detect, with 95% confidence, a 7% increase in compliance, in the hypothesis of 15% compliance in the control arm. Sampling started in Rome, with the very sample size as previously planned (800). As a result, extra self-sampler boxes were available for the study in Florence, in which the sample size was slightly increased (235 per arm=940 total). In Abruzzo, we later planned an additional fifth arm, slightly decreasing the number of women in the four arms (180 per arm). Actually the fifth arm was implemented as a separate trial, a few months after this study, owing to the earthquake that destroyed part of the Abruzzo region in April 2009.

### Description of the intervention

In the two control arms, the intervention was only the recall letter inviting for a Pap smear (arm (a)) or an HPV test (arm (b)) at a prefixed date at the clinic.

In the arm with the self-sampler at request, we mailed a letter inviting women to dial a free-toll number to receive the self-sampling device and an information leaflet on HPV test and cervical cancer. We offered women two opportunities: to either have the device shipped home or pick it up by themselves at the clinic. The self-sampler box contained the following items:

- a presentation letter;
- a leaflet about HPV test and cervical cancer prevention;
- the informed consent form to perform the test and to be contacted by phone in case of positivity;
- the self-sampler device;
- the instructions for the device use;
- a tube and a pre-paid envelope to mail of the sample; and
- a short questionnaire asking the approximate date of the latest Pap-test, questions about the sampling (if it was easy, annoying, embarrassing) and the date of sampling.

In the arm with the direct mailing, we sent an alerting letter explaining that the local Public Screening Program would provide women with a box containing a self-sampler device completely free. After 1 week, we shipped the box, containing the same items listed above.

Questionnaires slightly differed among centres owing to length constrains and local priorities. In this study, we analyse only the questions that were almost identical in the three versions: date of women's latest Pap-test, reason for non-compliance with the screening programme (only Rome and Florence), questions about the self-sampling performance (pain, embarrassment, feasibility), what was mostly appreciated in the self-sampling (doing it by themselves, privacy, absence of a doctor, absence of speculum), which kind of sampling was preferred (i.e., self-performed or carried out at the clinic).

### Management of HPV-positive women

All women who resulted in being HPV positive were contacted by phone and letter to propose a counselling on HPV and cervical cancer risk and a colposcopy. If the colposcopy was positive, a biopsy was taken, and if colposcopy was negative, women were suggested to undergo control with Pap-test within a year's time.

### Outcome

The main outcome we took into consideration was women's participation in screening: all women who accepted to perform a test in the screening programme within 3 months from the first letter mailing were considered a success.

A secondary outcome was the impact on cervical cancer screening coverage, measured as the number of women never-covered or under-covered (latest test performed longer than 3 years earlier) for whom we obtained a cervical sample.

Other secondary outcomes were HPV positivity rate and detection rate for CIN2+ in the intervention arm.

### Laboratory methods

#### Samples pretreatment

When samples arrived at the laboratory, they were centrifuged at 500 **g** for 10 min. Supernatants were discarded and pellets suspended in 1 ml of STM (Specimen Transport Medium; Qiagen, Hilden, Germany). Before performing Hr-Hybrid Capture 2 (HC2), 200 *μ*l aliquots were stored at −80°C.

#### HPV testing and evaluation of sample adequacy

Highrisk (HR) HPV was evaluated by HC (Qiagen), using only the B probe mix, which is specific for 12 HR HPV types: 16, 18, 31, 33, 35, 39, 45, 51, 52, 56, 58, 59 and one probably carcinogenic HPV type: 68 (Bouvard *et al*, 2009). The recommended positivity threshold of 1 pg ml^−1^ (equivalent to 5000 viral copies per test well) was used as a cutoff control value, and all samples with an RLU/Control ratio ⩾1.00 were considered HR-HPV positive.

In Florence, all HC2-negative samples were further analysed to evaluate sample adequacy. DNA was extracted from 200 *μ*l of un-denaturated STM samples with QIAamp DNAMini kit (Qiagen) according to the manufacturer's instruction. Amplification of the human *β-globin* gene was performed using GH20-PC04 primers (268-bp amplicon length) (Bauer *et al*, 1992). One HPV-negative sample was included in each batch of extraction to exclude any contamination. Polymerase chain reaction products were run on 2% ethidium bromide-stained agarose gels and visualised by ultraviolet light.

### Analysis

We adopted an intention-to-treat analysis: women were considered as participating, independently of the randomisation arm, if they provided any kind of sample (Pap smear, STM or self-collected liquid sample), in any setting (home or screening clinic).

We report the relative risk (RR) of having a test comparing each intervention arm with the two controls. Participating centres samples were pooled if there was no heterogeneity.

We used the information collected with questionnaires to calculate the impact on population coverage of each strategy according to the following formula: 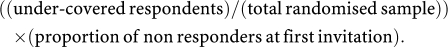


## Results

Nine hundred and fifty-one women were randomised in Florence, 800 in Rome and 729 in Abruzzo. Six women were excluded after randomisation in the direct mailing arm because the kit was not mailed owing to a logistical mistake and one was excluded because she had had a Pap-test in the screening programme before randomisation, but it had been notified to the coordinating centre just after the study started.

Table 1 shows the compliance by arm and by centre.

### Effect on compliance

The compliance with standard recall was 13.9%, similar to that obtained with a standard letter inviting women to perform HPV test at the clinic (14.9%). The group which received the self-sampler on demand had a poorer performance as compared with the standard recall letter arm, RR 0.62 (95% CI=0.45–0.86) with no differences among centres. The self-sampler direct mailing strategy had, on average, a higher performance and increased compliance by 40% (RR 1.41, 95% CI=1.10–1.82) with respect to standard recall, but it showed a border line significant heterogeneity among centres. In fact, the positive effect was detectable only in urban areas, Rome and Florence, whereas in Abruzzo, a prevalently rural, small town area, no positive effect was detected.

### Positivity rate and detection rate

In the direct mailing arm, according to the dates reported in the questionnaires, the median time elapsed from the sampling and the laboratory check in for the specimen was 4 days and only one sample took longer than 1 week (8 days). Nevertheless, in Abruzzo, where the kits were mailed in July with very high environmental temperatures, some specimens looked contaminated by a strong bacterial growth and two samples were unsatisfactory.

The overall positivity rate among the self-collected samples in the direct mail arm was 21.8% (22 out of 101). All but two women accepted to perform a colposcopy (91%) and no CIN2+ was found. The positivity rate in the HPV control arm was 6.5% (5 out of 77), and the difference was statistically significant (*P*=0.006).

The Pap-test ASCUS or more severe in the standard recall arm was one out of 86 (1.2%). The woman had a colposcopy and no CIN2+ was found.

### Acceptability of the self-sampling device

We collected 147 questionnaires. The most frequent reason for failing to comply with previous screening invitation was recent Pap-test (40.6%). No woman declared that Pap-test is not important and only two of them declared that they did not perform the test because they were embarrassed (Table 2A). Table 2B shows the answers about the sampling: 88.3% (128) of women declared that the sampling was easy, only two reported that it was annoying and two reported that it was embarrassing. The most checked reasons for which they appreciated the self-sampling were ‘to do the sampling by myself’ (57.6%) and ‘privacy’ (49.3%).

In the open answer questions, some women expressed concerns about the quality of self-collected samples, and six of them complained about broken devices (in all cases the sample was taken and analysed). No woman reported concerns about having the HPV viral test instead of the cytological test, nor about the way of infection.

### Impact on population coverage

Only six women (4.1%) referred to never having had a Pap-test before and five did not remember (3.4%). Among the others, 12 (8.2%) had had a test longer than 5 years before, and 45 (30.8%) had had it between 3 and 5 years before.

The impact on coverage was computed only for Florence and Rome, the very places where a definite gain in coverage has been proved; and for the arm in which an increase in compliance as compared with standard recall has been observed: the direct mailing arm. Out of 91 women responding to the direct mailing of the kit in Florence and Rome, four had never had a Pap-test before and 30 were under-covered, that is, they had undergone a test longer than 3 years earlier, whereas the rest of women had had a Pap-test within 3 years. The proportion of not- or under-covered women on the total randomised sample is 34 out of 438 (7.8%). This proportion must be applied to the population that had not responded to first invitation, that is, the eligible population for the trial. This proportion in Florence and Rome was 65%. According to the formula proposed in the Population and Methods section, the impact of direct mailing on population coverage would be: (34/438) × 0.65=+5.1% (95% CI=3.6–7.1). If we consider under-covered those who had had a Pap-test over 5 years earlier, the impact on compliance would then be 2.2% (95% CI=1.2–3.7).

## Discussion

Our results show that delivering self-samplers may increase compliance with cervical cancer screening programmes. Even if the observed heterogeneity was not statistically significant, we must highlight that a positive effect on compliance was observed only in Florence and Rome. Many context factors might explain this difference: there could be cultural and behavioural differences between urban and rural areas, or there may be more logistical problems in shipping self-sampler boxes in rural areas owing to different shape or location of the mail boxes. Finally, in Abruzzo the trial was performed in mid-July, when compliance with screening programmes is usually low and decreases as we get nearer August, when most Italian families are on holidays. In this period, the additional week needed in the direct mailing arm may have had a strong negative influence on compliance. This delay in performing our analysis was owing to the above-mentioned earthquake hitting the Abruzzo region in April 2009, which halted all studies for weeks.

We only found one randomised controlled trial comparing the effect of using the self-sampler instead of the standard recall letter for conventional cytology on compliance with screening invitation (Gök *et al*, 2010).

Some non-randomised trials showed a potential impact to increase test uptake and feasibility on large scale (Belinson *et al*, 2003; Salmerón *et al*, 2003; Sanner *et al*, 2009; Ogilvie *et al*, 2007; Szarewski *et al*, 2007). Two systematic reviews (Petignat *et al*, 2007; Stewart *et al*, 2007) concluded that the accuracy of self-sampling-based HPV test makes it suitable as an alternative mean for cervical screening, and one review affirms (Stewart *et al*, 2007) that there is lack of evidence on its impact on compliance.

Our trial design allowed us to distinguish the effect of offering a new test, that is, HPV instead of Pap, and the effect of self-sampling. In fact, we compared the intervention arm to two different control letters: the standard one, inviting for a Pap-test, and a similar letter inviting women to an HPV test with the same modalities. In Rome and Abruzzo, the two controls had the same performance, but in Florence, the HPV control had an intermediate performance between the standard Pap-test recall letter and the direct mailing of the self-sampler. Even if the observed differences are not statistically significant, the results in Florence may suggest that, at least in that context, part of the increase in compliance could be owing to the appeal of the new test. It should be taken into account that Florence was one of the participating centres in NTCC trial (Ronco *et al*, 2006). Consequently, the awareness of the population about HPV may be higher in this area.

Our study was not designed to detect differences in positivity or detection rate, but the difference in positivity rate between the control HPV and the direct mailing arm, as well as the low positivity in the Pap-test arm, suggests that women responding to self-sampling may be at higher risk of HPV infection. The difference may be also owing to the sampling technique or to the transport medium making HCII with self-sampling less specific than that performed at the clinic. Previous studies observed only small differences in positivity rate in double sampling or randomised studies (Belinson *et al*, 2003; Salmerón *et al*, 2003; Brink *et al*, 2006). On the other hand, a higher risk in non-responding women has been reported in several studies (Ogilvie *et al*, 2007). In particular, Gök *et al* (2010) found a higher risk in women responding to self-sampling.

We did not face important logistical problems. Few kits were returned late by the postal provider, and no claims for privacy or other individual offences came from the women involved. On the other hand, we had some evidence that very high external temperature may be a problem for sample conservation during transport through standard mail.

### Acceptability

The test was very well accepted by responding women, and the majority of them declared to prefer this way of sampling compared with the clinical sample. Pain and embarrassment were not problems at all, although some women declared it was not easy or that they were concerned about having collected the sample properly. The main reason to prefer self-sampling was the do-it-yourself opportunity given by the device, but also the less frequently checked answers, the absence of speculum and the absence of a doctor, were chosen by a relevant proportion of women. These results are consistent with previous studies (Anhang *et al*, 2005; Waller *et al*, 2006). The reasons for failing to comply with screening invitation confirmed what was already known for Italy: opportunistic screening outside screening programmes is very widespread and logistical barriers interfere with compliance (lack of time, failure to receive the letter).

### Impact on population coverage

In the self-sampling arms, the proportion of not- or under-covered women was quite high among those who accepted to perform the test: according to questionnaires, more than 43% of women had had a Pap longer than 3 years earlier. According to the National Health Interview and the PASSI survey (ISTAT, 2006; PASSI, 2009), in these areas the proportion of not- or under-covered (latest Pap-test >3 years before) women is much lower: 16% in Rome, 15% in Florence and 25% in Abruzzo. The difference is not surprising, as the eligible population in our study included only programme non-responders. The high Pap-test coverage in Florence and Rome is the effect of widespread opportunistic screening, both in the public and the private sector (Giorgi Rossi *et al*, 2006; ISTAT, 2006; PASSI, 2009). In this urban context, a 5% increase brings the coverage up to 90%. Considering that this estimate does not exclude virgins, and permanently sick women, and only partially accounts for hysterectomies, 90% may be considered a virtually full coverage.

Nevertheless, a 5% increase in coverage (2% if we only consider under-covered women with a Pap-test longer than 5 years earlier) is relevant in terms of public health. An increase in population Pap-test coverage of 1 or 2% is what has been obtained by most trials that measured the impact and not only the RR of the intervention *vs* control arms (Eaker *et al*, 2004; Corkrey *et al*, 2005; Jensen *et al*, 2009). The Danish Health Technology Assessment of cervical cancer found that an absolute increase in the coverage rate of 1% equals the risk reduction obtained by reducing the interval from 3 to 2 years and estimates a gain of 85 life-years each year in Denmark (Sundhedsstyrelsen *et al*, 2005).

If the absolute relevance of such a gain in population health is not questionable, the costs to obtain it must be carefully considered. In our trial, we had to send out five self-samplers to get one sample back to be tested. This number goes up to 13 if we consider the number needed to gain uncovered women to screening. Low response rate and high population coverage owing to overlapping of organised and opportunistic screening are context factors that minimise the advantages and maximise the costs of direct mailing: we must ship many self-samplers to non-responders, but only a few under-covered women are the real target of the intervention. We tried to reduce costs sending the samplers only to women who were interested and requested it, but this strategy had a worse participation rate than standard recall letters. In this trial, we did not investigate self-sampling strategies that might strongly reduce the workload for midwives, that is, not targeted only to non-responders, but also to all the target population.

### CONCLUSIONS

Self-sampling for HPV testing is acceptable to women and mailing a device for HPV DNA self-collection to non-responders to cervical cancer screening may increase compliance as compared with standard recall letters. Nevertheless, effectiveness is context specific. On top of this, the strategy is very expensive in terms of number of self-samplers needed per screened woman.

## Figures and Tables

**Table 1:** Compliance by arm and type of test

**Intervention**	**Invited women**	**Test HPV**	**Pap-test**	**Compliance (%)**	**RR *vs* Pap-test**	**95% CI**	**Test for heterogeneity**	**RR *vs* HPV**	**95% CI**	**Test for heterogeneity**
*Total*										
Standard recall	619	0	86	13.9	1			0.93	(0.71–1.22)	0.27
Self-sampling on demand	622	36	18	8.7	**0.62**	**(0.45–0.86)**	0.92	**0.58**	**(0.42–0.80)**	0.22
Self-sampling direct mailing	616	103	18	19.6	**1.41**	**(1.10–1.82)**	0.11	1.32	(1.03–1.68)	0.01
HPV at the clinic	616	77	15	14.9	1.08	(0.82–1.41)	0.27	1		
										
*Florence*										
Standard recall	238	0	34	14.3	1			0.76	(0.50–1.14)	
Self-sampling on demand	240	12	9	8.8	0.61	(0.37–1.02)		**0.47**	**(0.29–0.76)**	
Self-sampling direct mailing	238	45	8	22.3	**1.56**	**(1.05–2.31)**		1.18	(0.83–1.69)	
HPV at the clinic	234	40	4	18.8	1.32	(0.87–1.98)		1		
										
*Rome*										
Standard recall	200	0	20	10.0	1			1.43	(0.74–2.75)	
Self-sampling on demand	200	12	2	7.0	0.70	(0.36–1.35)		1.0	(0.49–2.04)	
Self-sampling direct mailing	200	36	2	19.0	**1.90**	**(1.15–3.15)**		**2.71**	**(1.52–4.85)**	
HPV at the clinic	200	14	0	7.0	0.70	(0.36–1.35)		1		
										
*Abruzzo*										
Standard recall	181	0	32	17.7	1			0.95	(0.61–1.46)	
Self-sampling on demand	182	12	7	10.4	**0.59**	**(0.35–1.00)**		**0.56**	**(0.33–0.94)**	
Self-sampling direct mailing	178	22	8	16.9	0.95	(0.61–1.50)		0.90	(0.58–1.41)	
HPV at the clinic	182	23	11	18.7	1.06	(0.68–1.64)		1		

Abbreviations: CI=confidence interval; HPV=human papillomavirus; RR=relative risk. Values given in bold indicate that RR is significantly different from 1, *P*<0.05.

**Table 2:** Answer to questionnaires

**(A) Reasons for non-compliance with screening programme invitation letter**		
**Reasons for non-compliance**	***N***	**%**
Recent Pap-test	41	40.6
No time	23	22.8
I did not receive the letter	15	14.9
I was out	7	6.9
I was pregnant	6	5.9
It is embarrassing	2	2.0
I think it is not useful	0	0.0
Other	7	6.9
Total	101	100

**(B) Attitudes to self-sampling**				
	**Yes**		**Total**	
	***N***	**%**	***N***	
*The sampling was*				
Easy	128	88.3	145	
Annoying	2	1.4	145	
Embarrassing	2	1.4	145	
I was not sure I had a good sample	17	11.8	144	
				
*I appreciated*				
To do the sampling by myself	83	57.6	144	
Not to be undressed in front of a doctor	37	25.2	147	
The absence of speculum	45	31.5	143	
Privacy	70	49.3	142	
				
	**Self**		**Clinic**	
**Which sampling method do you prefer**	***N***	**%**	***N***	**%**
	109	78.4	30	21.6

## APPENDIX

**Members of the Self-Sampling Study working group:**

Rome: Paolo Giorgi Rossi, Alessandra Sperati, Laura Camilloni, Laila Maria Marsili, Paola Capparucci, Concetta Bellanova and Piero Borgia; Abruzzo: Claudio Angeloni, Amedeo Lattanzi, Tatiana Reggi, Patrizia Bruni and Clara Monaco; Florence: Anna Iossa, Massimo Confortini, Francesca Maria Carozzi, Elena Burroni, Giovanni Pontenani, Carmen Beatriz Visioli, Cristina Sani, Carmelina Di Pierro and Elisa Sereno.
